# A re-assessment of *Taxomyces andreanae*, the alleged taxol-producing fungus, using comparative genomics

**DOI:** 10.1186/s43008-022-00103-4

**Published:** 2022-09-26

**Authors:** Tian Cheng, Miroslav Kolařík, Luis Quijada, Marc Stadler

**Affiliations:** 1 Department Microbial Drugs, Helmholtz Centre for Infection Research (HZI), Inhoffenstraße 7, 38124 Braunschweig, Germany; 2 German Centre for Infection Research (DZIF), Partner Site Hannover-Braunschweig, Inhoffenstraße 7, 38124 Braunschweig, Germany; 3 Institute of Microbiology of the ASCR, v.v.i., Vídeňská 1083, 14220 Prague, Czech Republic; 4 Department of Organismic and Evolutionary Biology, The Farlow Reference Library and Herbarium of Cryptogamic Botany, Harvard University, 22 Divinity Avenue, Cambridge, MA 02138 USA; 5 Institute of Microbiology, Technische Universität Braunschweig, Spielmannstraße 7, 38106 Braunschweig, Germany

**Keywords:** *Ceriporiopsis*, Endophytic fungi, Genome mining, Phylogeny, New taxon

## Abstract

The monotypic “bulbilliferous hyphomycete” genus *Taxomyces* was erected in 1993 for a fungal endophyte isolated from the Yew tree *Taxus brevifolia* and named *Taxomyces andreanae.* This fungus was reported to produce the plant-derived anti-cancer drug taxol. The original description of the fungus was not conclusive as to its taxonomic position because no sporulation or other salient morphological features were reported. Consequently, the taxonomic affinities of this fungus have remained obscure. However, a full genome sequence of this strain was generated by a German research group in 2013, in an unsuccessful attempt to detect the biosynthesis genes encoding for taxol. This prompted us to search for phylogenetic marker genes and compare those with the data that recently have become available from state-of-the-art polyphasic taxonomic studies. Surprisingly, the strain turned out to belong to the phlebioid clade of wood-destroying *Basidiomycota* as inferred from a comparison of its partial ITS, the 28S rDNA (LSU), the RNA polymerase II largest subunit (rpb1), the RNA polymerase II second largest subunit (rpb2), and the translation elongation factor 1-α (tef1) sequences. A multi gene genealogy based on these loci revealed that the closest relative is *Ceriporiopsis* (syn. *Mycoacia*) *gilvescens*. Even though such wood-destroying *Basidiomycota* are regularly encountered among the endophytic isolates after surface-disinfection of plant organs, the vast majority of the reported endophytic fungi belong to the *Ascomycota*. Nevertheless, the data available now allow for synonymizing *Taxomyces* with *Ceriporiopsis*, and the necessary new combination is made.

**Supplementary Information:**

The online version contains supplementary material available at 10.1186/s43008-022-00103-4.

## Introduction

Fungal taxonomy has changed drastically throughout the past decades, owing to the advent of molecular methods, and now it is even possible to use full genome data in order to define boundaries between species, genera and higher taxa (Lücking et al. 2020). The situation in the last century was much different, and it was next to impossible to assign environmental isolates to a certain taxonomic group when they did not produce any sporulating structures. Such “sterile mycelia” are frequently being encountered among the endophytes of seed plants that were isolated after surface disinfection of plant tissues. Among the most famous of these endophytic hyphomycetes was *Taxomyces andreanae*, a fungus that was reported to be able to produce the plant-derived anticancer agent, taxol by Stierle et al. (1993). Concurrently to the report of the production of this terpenoid, Strobel et al. (1993) erected a new monotypic genus of hyphomycetes to accommodate this fungus. Those authors did not find conidia or other salient discriminatory morphological features, except for some non-germinating mycelial clumps that they characterized as “bulbils”. It was not possible at that time to assign the strain to a higher taxon using molecular phylogenetic evidence and until now, the taxonomic position of the genus has not been clarified.

Heinig et al. (2013) obtained the ex-type culture from CBS (now Westerdijk Fungal Biodiversity Institute, Utrecht), where it was deposited under the Budapest Treaty in the course of a corresponding patent application under the accession no. CBS 279.92. They obtained a full genome sequence and attempted to find the biosynthetic genes encoding for taxol production, but failed to find any using genome mining. In the course of our ongoing work on the phylogenomics of fungi (Stadler et al. 2020; Wibberg et al. 2021), we have found that phylogenetically important barcoding loci can often be retrieved from the full genomes, provided that the data quality is sufficient. Therefore, we checked the genome data available from the study by Heinig et al. (2013) for genetic barcodes and here report on the phylogenetic and taxonomic position of *Taxomyces* for the first time.

## Material and methods

### Genome of *Taxomyces andreanae*

The genome information of *Taxomyces andreanae* CBS 279.92 was obtained from GenBank (reference no. ASM196922v1).

### Identification of ITS, 28S, rpb1, rpb2 and tef1 sequences

The genome sequence of *Taxomyces andreanae* CBS 279.92 was used to create a BLAST database in Geneious 7.1.9 (https://www.geneious.com). Previously published ITS, 28S, rpb1, rpb2 and tef1 sequences in GenBank were used as template for a homology search to locate each region.

### Alignment and phylogenetic analyses

A dataset of combined ITS + 28S + rpb1 + rpb2 + tef1 was analyzed to confirm the generic placement of *Taxomyces andreanae.* All alignments were made with MAFFT v. 7.313 (Katoh and Standley, 2013), using the default algorithm. The evolutionary history of both, the ITS sequences and the combined dataset was inferred by using the Maximum Likelihood method and Tamura-Nei model. Initial trees were obtained automatically by applying Neighbor-Join and BioNJ algorithms to a matrix of pairwise distances estimated using the Tamura-Nei model. A discrete Gamma distribution was used to model evolutionary rate differences among sites [5 categories (+ G)]. Phylogenetic analyses were conducted in MEGA11 (Tamura et al. 2021). All sequence data that we used for the analysis are provided in the Additional file 1.

### Revision of the type specimen

The type of *Taxonomyces andreanae* deposited in the FH herbarium was examined to re-assess the morphology of the fungus (Fig. 3). The surface of one branch was scraped off with a needle to get a small portion of the fungal mycelium and then pretreated with KOH, after that it was mounted in aqueous Congo Red or Melzer’s reagent, respectively, to contrast cells walls or analyse reactions. The pictures were taken with a Moticam USB cam attached to a Motic BA310E trinocular microscope.

## Results

**Fig. 1:**
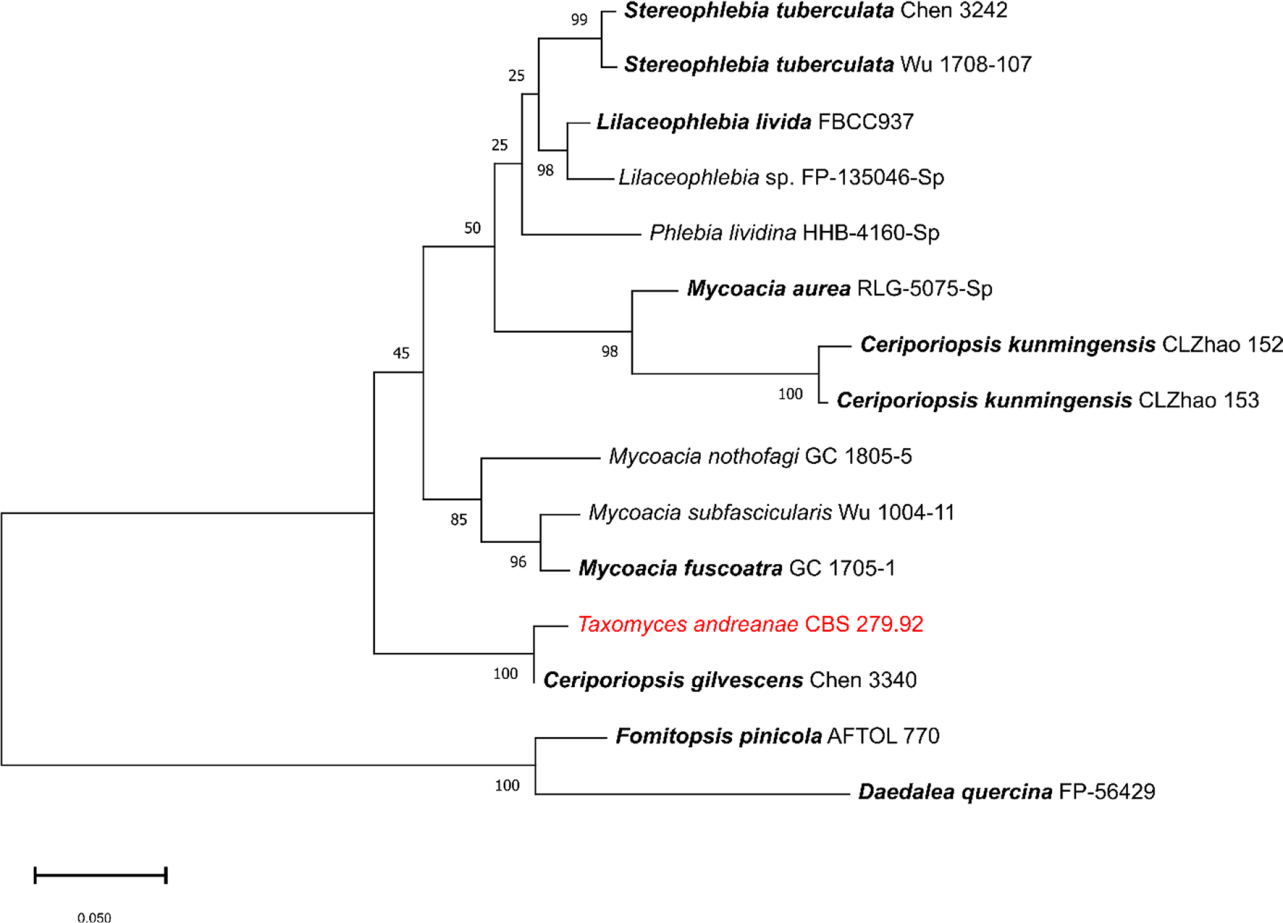
Phylogram of *Taxomyces andreanae* and phlebioid species inferred from ML analyses using the combined ITS sequences retrieved from a BLAST search. The type species of the respective genera are printed in bold, even though most sequences are not derived from type or epitype material

From the genome data, we have been able to retrieve several fragments relating to important marker genes that are used in fungal phylogeny, starting with the ITS. The only incomplete ITS sequence that was retrieved from the genome data of *T. andreanae* showed a high homology to the sequence of *Ceriporiopsis gilvescens* published by Chen et al (2021). The other most homologous ITS sequences were also derived from members of the phlebioid clade of *Basidiomycota* (Fig. 1). Interestingly, all of these fungi are known to be saprotrophic wood-destroyers, even though it is of course possible that they occur as endophytes in healthy plants (see Stone et al. 2000 for an overview). After all, the horizontally transmitted endophytes do not reside permanently in their plant hosts, but many of them are known to have a saprotrophic and an endophytic life stage. For instance, this applies to most species of *Hypoxylaceae* and other genera of *Xylariales*, whose saprotrophic stages are frequently encountered on dead wood as well (cf. Becker and Stadler 2021).

Based on the BlastN search against GenBank, the ITS and LSU loci showed high similarity with sequences of *Ceriporiopsis gilvescens* originating from Europe, USA and East Asia, many of which originated from reliable taxonomic studies. The ITS sequence containing the ITS1 spacer only was identical with entry KJ668562 (Jang et al. 2016, South Korea), HQ659222 (Miettinen and Rajchenberg 2012, Czech R.), FJ496684 (Tomšovský et al. 2010, Czech R.), MZ636935 (Chen et al. 2021, Taiwan) but showed 1 bp difference from KJ140684 (USA, Brazee et al. 2014). The LSU sequence was identical with FJ496720, FJ496721 (Tomšovský et al. 2010, Czech R.) and showed 1 bp difference to MZ63709 (Chen et al. 2021, Taiwan). The tef1 gene was 97.91% similar with the entry MZ913651 (Chen et al. 2021, Taiwan) and the rpb2 sequence was 98.34% similar to OK136039 (Chen et al. 2021, Taiwan). The similarity of the rDNA barcodes suggests that the CBS strain is closely related or even identical with the species *Ceriporiopsis gilvescens.* This species is found throughout the northern hemisphere (cf. https://www.gbif.org/species/2541778) and its presence in Montana is therefore possible. However, the differences observed in the tef1 and rpb2 genes indicate that it could just as well be a related species. The absence of fruitbodies makes further comparisons difficult, and the taxonomy of *C. gilvescens* clearly needs revision using multiple specimens and genes. We therefore leave the position of *Taxomyces andreanae* unresolved for now, but propose its combination into the genus *Ceriporiopsis* (see below).

**Fig. 2:**
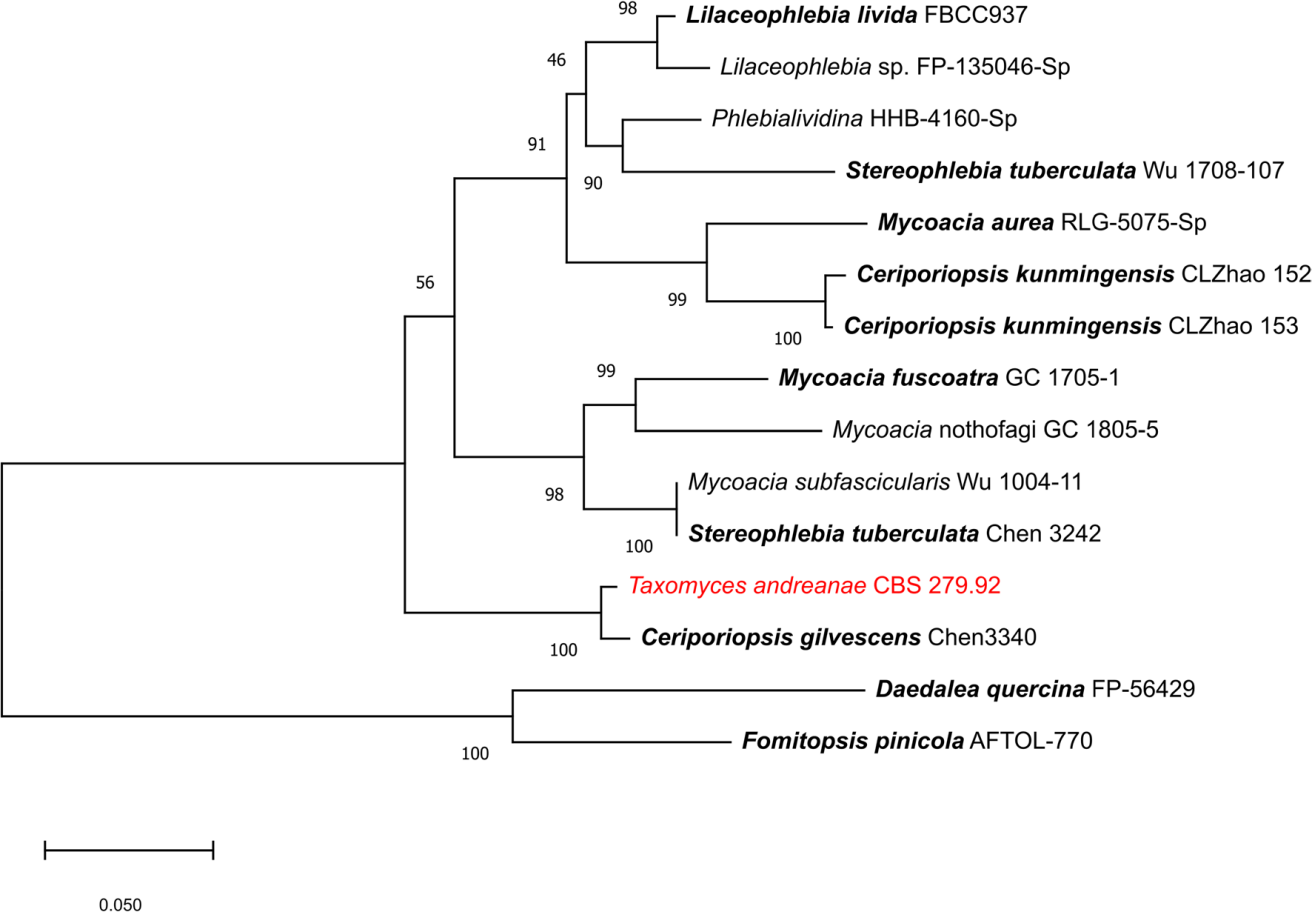
Phylogram of *Taxomyces andreanae* and phlebioid species inferred from ML analyses using the combined ITS + LSU + rpb1 + rpb2 + tef1 dataset. Sequences are listed in the Additional file 1. The type species of the respective genera are printed in bold, even though most sequences are not derived from type or epitype material

Next we used the data from Chen et al. (2021), who recently published a multi-locus genealogy including many representatives of the phlebioid clade, and incorporated the sequences from the *Taxomyces* genome. As shown in Fig. 2, the inclusion of protein-coding genes led to a substantial stabilization of the phylogeny. *Taxomyces* was located in the clade with *Ceriporiopsis gilvescens* “Chen 344”, while the topology of the tree was otherwise the same as in the original publication. According to Chen et al. (2021) who did not give details of the specimen collection data, the material they took for their study was collected either from Taiwan or “borrowed” from institutions in mainland China or USA. Therefore, some doubt remains on the identity of the *C. gilvescens* voucher they used in that study. Many other sequences derived from vouchers collected in Europe, however, showed a rather high homology to the sequences generated by Chen et al. (2021). Among those were ones conducted by specialists on these fungi, such as FJ496684 from the study of Tomšovský et al. (2010).

The work by Chen et al. (2021) is the most conclusive phylogenetic study on these fungi yet available. It showed that species assigned to *Ceriporiopsis* and the related *Mycoacia* do not form a monophyletic group, and we agree with their decision to leave the type species of *Ceriporiopsis* in that genus and reject the proposal by Zmitrovich (2018) to merge it with *Mycoacia*. The type species of the latter genus, *M. fuscoatra,* appears in a sister clade in Figs. 1 and 2. *Ceriporiopsis gilvescens* was first described by Bresadola (1908), based on material collected in France and originally named as *Poria gilvescens*; that name does not appear to have been epitypified by sequenced material from the original location. That species is, however, the type species of *Ceriporiopsis* (Domanski 1963), and so any further taxonomic change in the placement of that fungus could well impact phylogenetically close relatives such as *Taxomyces*.

The phylogenetic analyses of loci derived from the genome suggest that *Taxomyces* belongs in the basidiomycete genus *Ceriporiopsis.* The necessary new combination is therefore made here.

## Taxonomy

***Ceriporiopsis andreanae*** (Strobel et al*.*) T. Cheng & M. Stadler, **comb. nov.**

MycoBank no.: MB845185

Figure 3

**Fig. 3:**
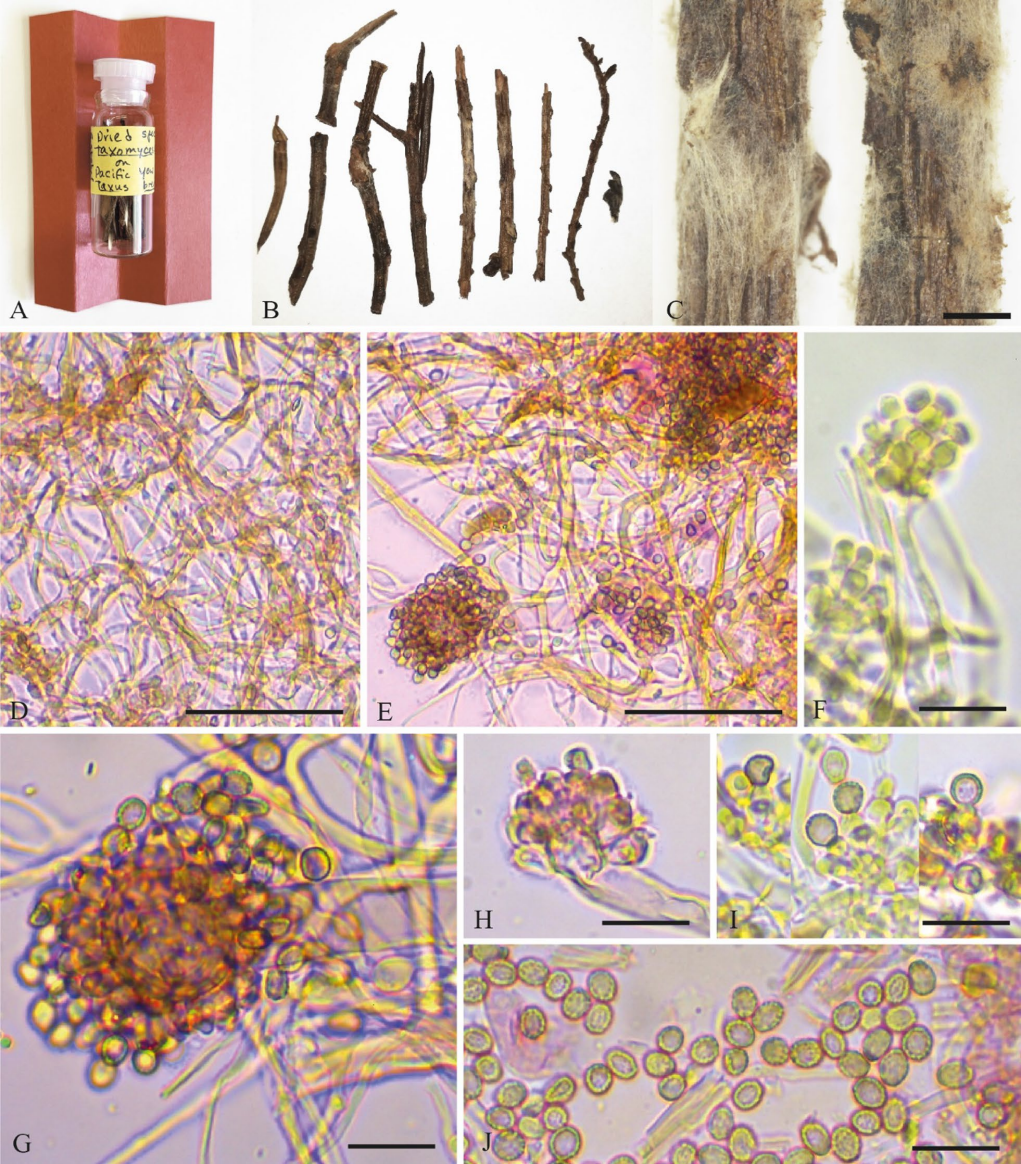
Morphology of the type of *Taxomyces andreanae*. **A**–**C**: plant twigs; C-M microphotograps. Scales: **C** = 1 mm, **D**–**E**. = 50 µm, **F**–**J** = 10 µm. Reagents: KOH + Congo Red = **D**–**E**, **G**, **H**, **J**; KOH + MLZ = **F**, **I**

*Basionym*: *Taxomyces andreanae* Strobel et al*.*, *Mycotaxon* **47**: 73 (1993).

*Type:* **USA**: *Montana*: Flathead Co., from the bark of *Taxus brevifolia*, Aug. 1991, *A. & D. Stierle* (FH—holotype; CBS 279.92, MSU 738—ex-type cultures).

The holotype in FH comprises: (1) a glass vial with bark fragments dated 19 Jan 1993, i.e. before the paper was published as the *Mycotaxon* volume was issued on 21 June 1993; (2) two SEM micrographs by W. M. Hess, which would have been obtained from cultures as there is no reference to the fungus being seen growing on the bark fragments; (3) a handwritten label by G. Strobel stating that the material was from “small limbs of *Taxus brevifolia* collected and produced by Gary Strobel” and that the SEMs were by Hess; and (4) a typed Farlow Herbarium label. Our revision of the holotype agreed with Strobel et al. (1993), but we found some morphological features not mentioned in their description. For example, the bulbil cells do not remain colorless (Fig. 3G–J), they are hyaline when immature but then change to light brownish (dark brownish in mass) when mature. The wall cells are slightly thickened up to 0.5 µm, Strobel et al. (1993) mentioned the bilayered cell wall, but not that the walls were ornamented with small spines which is clearly visible in our study (Fig. 3J). Also, we found biometric differences in the measures of the bulbil cells. According to our measurements they are longer and wider, (2.1)3.1–3.5(4) × (1.7)2.5–3.5 µm versus 1.5 × 2.5 µm as reported by Strobel et al. (1993) description.

No mention of Flathead County nor of the 1991 date appear on any of these materials. Strobel et al. (1993), however, carried out experiments inoculating the endophyte onto twigs, leaves or bark of eight different tree species, including *T. brevifolia* where the endophyte production of bulbils was said to be “heavy”. It is therefore possible that the “collected and produced by” comment indicates that these were from the inoculation experiments that had been carried out. Intriguingly, we also note that the original account says “Holotypus: Based on material taken from the bark of *Taxus brevifolia*.... August 1991... Flathead County”. This supports our suspicion that the material deposited in FH was most likely to have been from an experiment using the isolate from 1991 and not the sample from which the endophyte was first isolated.

We must also draw attention to the statement “ = *Cladorrhinum*.. *fide* Gams” in Seifert et al. (2011: 434), but with no further explanation and no formal transfer of the epithet into *Cladorhinum*. We suspect that this remark was based on the illustrations where there are some similarities in the clumps of conidia in *C. foecundissimum* to the “bulbils” of *Taxomyces*. There is no annotation slip to suggest he ever examined the material in FH, and neither is there a mention of the CBS culture being examined. Considering the vast experience of Walter Gams, we suspect this was a most likely an unfortunate quick decision made when rushing to complete the Seifert et al. work which could well have been influenced by Strobel et al. (1993: 74) suggesting a relationship to that genus.

The genomic data we have retrieved are much better in accordance with the protologue by Strobel et al. (1993). Morover, cladorrhinum-like conidiophores (typical of a dematiaceous hyphomycete) are very different. Indeed, *Cladorrhinum* is actually a member of the *Sordariales*, it is currently placed in *Podosporaceae* (Marin-Felix and Miller 2022).

We are confident that Heinig et al. (2013) studied the correct fungus, as this was deposited in CBS in 1992 under the special procedures, required by the Budapest Treaty and Regulations 1981 for deposits related to patent applications, in this case regarding the fungal taxol production, so particular care would have been taken to ensure the correct fungus and no contaminant was deposited. We are unsure of the practice at CBS at that time, but the leading culture collections would generally ask depositors to check the identity of the deposited strain after preservation in their collection.

## Discussion

This study shows the power of genome sequencing to resolve the taxonomy of fungi that cannot easily be classified based on morphology alone. Complete genome sequences cannot only be mined for phylogenetic marker genes, but even for other genes that encode, e.g., for secondary metabolite biosynthesis (Kuhnert et al. 2021). The fact that we were able to retrieve all the salient marker genes that are presently used for phylogenetic classification in the phlebioid *Basidiomycota* also proves that the quality of the genome sequence was good enough, even though the Illumina technique used by Heinig et al. (2013) resulted in many contigs. We did not attempt to re-do their work and try to detect biosynthesis genes encoding for secondary metabolites (which goes far beyond the scope of the current study), but are sure they would have found the taxadiene synthase from the plant if it had been present.

The original description of the species does not offer many options to determine whether it belongs to *Basidiomycota* or *Ascomycota*, and some differences between the original diagnosis and our own study were noted, regarding the bulbil color and wall ornamentation (Fig. 3). The cultural characters of *C. gilvescens* are unknown, but the description of *Taxomyces* and our own observations of the type are not in conflict with the characteristics of the mycelial cultures of the related genus *Phlebia*, only to give an example. These features are: the presence of hyaline mycelium without clamp-connections (in the case of primary mycelium), multinucleate hyphal cells and bulbils formation. Indeed, it is the formation of bulbils bearing conidia-like projectiles, which do not germinate, that can be found in various *Polyporales* (Stalpers 1978), but according to our knowledge not in ascomycetes. Strobel et al. (1993) also mentioned the absence of dolipores and presented images of ultramicroscopic structure of conidia-like cells from bulbils, but unfortunately, cellular septa were not depicted.

The current case, where a culture of the type material has been deposited in a public domain collection from where it can be obtained later on for genome sequencing, is also a very good example for the validity of the approach by Yurkov et al. (2021). Among others, these authors have emphasized the need to use living cultures as type material in the future. If this had been possible in case of *Taxomyces*, some ambiguity as to the taxonomic position of the fungus could perhaps have been avoided.

That we were able to determine *Taxomyces* was a basidiomycete raises the question of the validity of all the previous studies where taxol production is reported from endophytic fungi that are exclusively from the *Ascomycota*. The rationale that endophytes of *Taxus* may have acquired the ability to produce this highly complex meroterpenoid during a long co-evolution of the fungi and their plant host may bear some merit. Still, it is difficult to envisage how many tentative studies have been published about this phenomenon. In the current context, it is impossible to deal with all the questionable scientific publications that have been published throughout the past decades. Therefore, we only will give some striking examples.

An historical overview of the reports on fungal taxol production shows that initially only endophytes of *Taxus* species have been studied (cf. Zhou et al. 2010; Garyali et al. 2013), which would have made sense considering the hypothesis of a horizontal gene transfer between host plant and the endophytes. Later on, the compound was reported from endophytes inhabiting a wide range of plant species, including other gymnosperms (e.g. Sentil Kumaran et al. 2008) and even aquatic and tropical angiospermous medicinal plants (Gangadevi and Muthumary, 2008; Pandi et al. 2011). Even without having a closer look at the analytical methodology used in the respective studies, these reports appear rather suspicious because, taken together, they would suggest that taxol was a primary metabolite. The fact that some of these papers were published rather recently (ignoring the evidence that has accumulated on the genetics of secondary metabolite biosynthesis) causes us to question whether the reviewers and editors of the respective journals have had the necessary level of expertise to rigorously assess the submissions.

There is now much hard evidence on the evolution of secondary metabolism, and especially the production of complex molecules and their corresponding biosynthetic genes in fungi (Keller 2019). Therefore, it is almost inconceivable that a molecule like taxol could have arisen convergently in *Ascomycota*, *Basidiomycota,* and plants. Taxol formally constitutes a meroterpenoid, which consists of a terpenoid backbone to which an amino acid is attached. Heinig et al. (2013) have already discussed that the taxol biosynthetic genes are not clustered in the genomes of the Yew plants, and so any horizontal gene transfer is difficult to envisage. Even almost 30 years after the report of taxol from a fungus, where the compound has been only detected in traces, we are not aware of any sustainable production process for this anticancer agent that uses a fungal culture. For instance, Yang et al. (2014) reported the isolation of very small quantities of taxol from a *Penicillium* sp. They characterized the compound by HPLC–MS and claimed they even measured nuclear magnetic resonance (NMR) spectra (but such data are not included). They postulated a convergent pathway for taxol biosynthesis from the genome data. However, there is no follow-up study to confirm the hypothesis of Yang et al. (2014) by state of the art methodology like heterologous production or generation of KO mutants. On the other hand, taxol derivatives are being produced sustainably using plant cell cultures or semi-synthetically from precursors that are extracted from the needles of the European yew, *Taxus baccata*, in kg scale (Holton et al 1995; Expósito et al. 2009; Malik et al. 2011). We therefore conclude that the aforementioned reports may have been due to the use of unsuitable analytical methods, and that taxol production is a specific feature of the *Taxus* plants, not of any fungus, but it is hard to prove a negative.

## Conclusion

This study has shown that it is possible to retrieve phylogenetic barcodes from a genome sequence that was created using the Illumina shotgun technique from an environmental isolate. We recommend using similar approaches as well to classify the numerous fungal strains that have been subjected to genome sequencing and are deposited in GenBank and other repositories but were not yet classified to species level. As recently outlined by Houbraken et al. (2021), it will be better to rely on multiple loci rather than on a single barcode sequence, in order to allow for a concise molecular identification.

## Supplementary Information

**Additional file 1.** Alignment of sequence data retrieved from the genome sequence generated by Heinig et al. (2013), corresponding sequence data and reference sequences that were used for the phylogenetic comparison (Table S1).

## Data Availability

Not applicable (no new data were generated).
