# Supplementary material for: A re-assessment of Taxomyces andreanae, the alleged taxol-producing fungus, using comparative genomics
Source: IMA Fungus. 2022 Sep 26;13:17. doi: 10.1186/s43008-022-00103-4 (PMC9511726; doi:10.1186/s43008-022-00103-4)
Supplement: Supplementary file 1 — Additional file 1. Alignment of sequence data retrieved from the genome sequence generated by Heinig et al. (2013), corresponding sequence data and reference sequences that were used for the phylogenetic comparison (Table S1). [file 43008_2022_103_MOESM1_ESM.pdf]

Supplementary Information for

A re-assessment of *Taxomyces andreanae*, the alleged taxol-producing fungus, using comparative genomics

Tian Cheng[1,2], Miroslav Kolarik[2] , Luis Quijada[3] and Marc Stadler[1,4]*

[1] Department Microbial Drugs, Helmholtz Centre for Infection Research (HZI), and German Centre for Infection Research (DZIF), partner site Hannover-Braunschweig, Inhoffenstraße 7, 38124 Braunschweig, Germany

[2] Institute of Microbiology of the ASCR, v.v.i., Vídeňská 1083, 14220 Praha, Czech Republic

[3] Department of Organismic and Evolutionary Biology, The Farlow Reference Library and Herbarium of Cryptogamic Botany, Harvard University, 22 Divinity Avenue, Cambridge, MA 02138, USA

[4] Institute of Microbiology, Technische Universität Braunschweig, Spielmannstraße 7, 38106 Braunschweig, Germany

*Corresponding author: marc.stadler@helmholtz-hzi.de

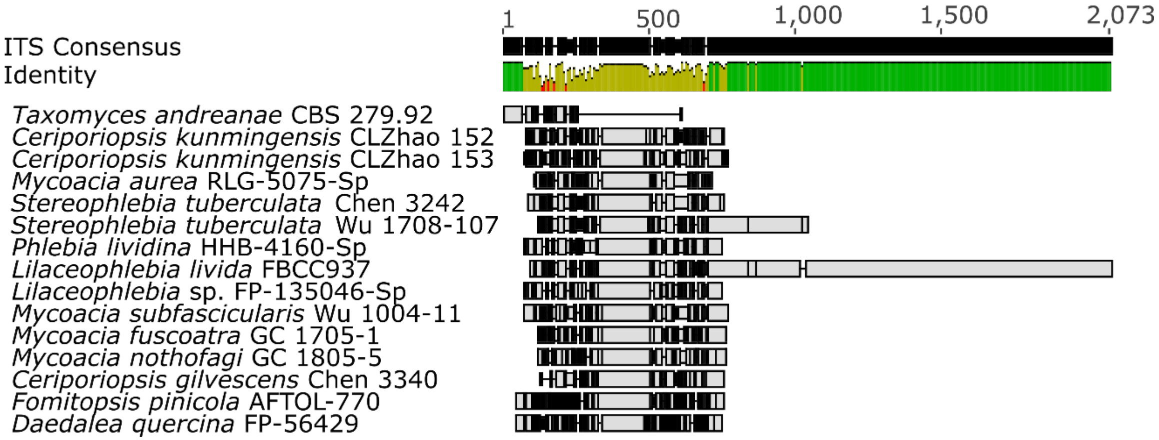

Fig. S1 Alignment of the ITS sequences.

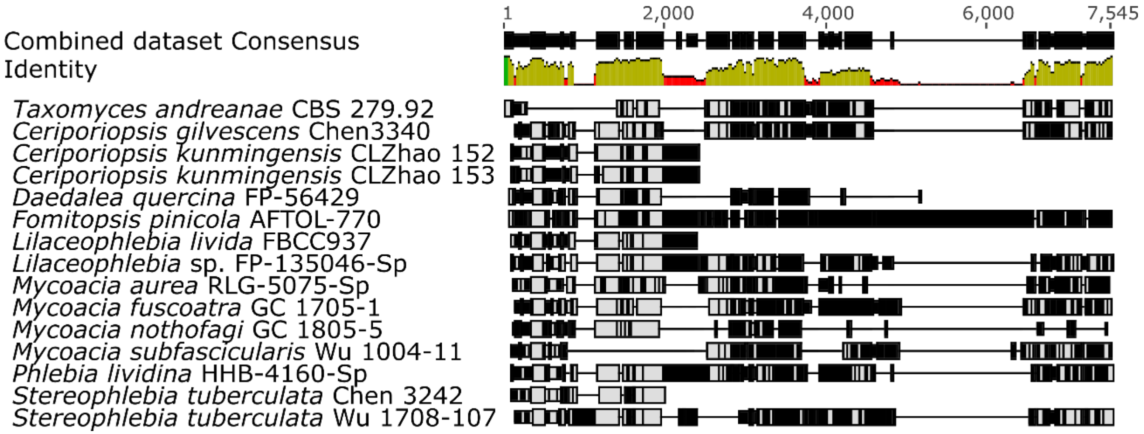

Fig. S2: Alignment of combined multilocus dataset.

Sequences of phylogenetic markers retrieved from the genome sequence

>*T. andreanae*-ITS (ALYI01000630.1)

GGCACCTTGTTGCTGAGAACTTGATCAAACTTGGTCATTTAGAGGAAGTAAAAGTCGTAACAAGGTTTCCGTAGGTGAACCTGCGGAAGGATCATTAACGAAATAACAAGGA
GTTGTTGCATGCCAAAATGGTGTCGCACGCTCTGTCCATTTCAAACCCCTGTGCACCTATTGTAGGCATGGTCGTAGGGCTGACTTCGGTCGGTTCGAAAGCC

> *T. andreanae*-28S

TTAAACAGTACGTGAAATTGCTGAAAGGGAAACGCTTGAAGTCAGTCGCGTCGTCCAGAACTCAACCTGGCTTTTGCTTGGTGCACTTTCTGAACGACGGGCCAACATCAGTT
TTGACTGTCGGAAAAAGTCCTTAGGAATGTGGCACCTCCGGGTGTGTTATAGCCTTTGGTCGTATACGATGGTTGGGACTGAGGACCGCAGCACGCCTTATGGCCGGGGTTC
GCCCACGTACGTGCTTAGGATGTTGGCGTAATGGCTTTAAGCGACCCGTCTTGAAACACGGACCAAGGAGTCTAACATGCCTGCGAGTGTTTGGGTGGAAAACCCGAGCGCG
CAATGAAAGTGAAAATTGGGATCCCTGTCGTGGGGAGCACCGATGCCCATACCAGACCTTCTGTGACGGATATGCGGTTGAGCATGTATGTTGGGACCCGAAAGATGGTGAA
CTATGCCTGAATAGGGTGAAGCCAGAGGAAACTCTGGTGGAGGCTCGTAGCGATTCTGACGTGCAAATCGATCGTCAAATTTGGGTATAGGGGCGAAAGACTAATCGACCAT
CTAGTAGCTGGTTCCTGCCGA

> *T. andreanae*-rpb1

AGGCTTCATCGTGAAAGTGAAGAAGATACTGGAATGTATATGCGTAAATTGTGGGAGACTGAAAGCCGACATCGTGAGTCGCGTCTCCCTTTCCTCTACTCGTTTTGCACCCCA
CCACCCAACTTTCTGGGGGCTCTCTCCGAACCCATGGAGAACCCCAAGCCCGCTCCGCGTTCGTTCTCGACAATCGAGGCGTTCTTCCACCCGTGACGTGGATCCCGCGCGTTT
TCGGTCGCTTGATCGCCGCGGGGTCCATTGCATAACGTCCGGTGTTGGTTGGACACGCGGTTGCGTGGTGAAATGGGGGGCTGAGCGAGGAGTGCGGGAAAATGCATCCAC
GGCATGGTCAAGCCATGCCGGGATATGTGGCCAAACTGCCCTCGCTGTGTCGCAGCTTGAGCGGCATGTTTTCGGCCTGCACTTAATACTCGATATCGTTATGCCCCCTGTTGT
TGCTCCGTTCCTTCCTGTGTCGTTGGTCGTGGAAGCAGTTTGCTGGAGTCCAGTCGCACATGCTGACGTTCTACCTTTTATGTTCGCCCCTACTCGCCGTATTCAACGCACGTGT
GAATTCGCATCGTAAGTCGGACCCTGCCTTTGCGGATAAAATTAGGCACGTTCGCGATCCGAAAGCTCGGATGCAAGTCGTGTGGAATTTCTGCAAGAGCAAAATGATTTGCG
AACCCGACGAGCCCAAGGACGAAAACGACAACGACGCTGAGGAGCCAAAGAAGGGCCACGGGGGCTGCGGCGCAGCACAGCCACAGATCAGGAAGGAGGGTCTGAAGCT
CTTCGTCCAGTACAAGCGGTCGAAGGATGAGGATGAGGTAGGTTTTGCCGCATATACGCTCACTGCACCGATCTTATAGCGCATGACAGGAGGTGAAGACGCTCCAGCCCGA
CAAGCGGCTCTTCCCACCTCACGAAGTGTACACGGCGCTGAAGAAGATCCCTGACTCCGATCTTCACTTACTCGGCCTCTCCGACGAGTACGCGCGCCCAGAATGGATGATTCT
CACTGTTCTGCCCGTTCCACCTCCGCCCGTGCGTCCAAGTATAGCGGTGGACGGCGGTACCATGAGAAGTGAGGATGATTTGACGTATAAGCTGGGAGATATCATCAAGGCG
TCTGCGAACGTCCGCAGATGCGAGCAAGAAGGCGCGCCGTCGCACGTCATCACCGAATTCGAGCAGCTGCTACAGGTGGGATTACGGTGACGGTTC

> *T. andreanae*-rpb2

GTTGACGAAGGATGTTTACCGTTACCTGCAGAAGGTGTGTATCAAAGTGAGATTCTTCACATTTGCAGACTCATTGTTATTTGCGTAGTGTGTGGAGACGCACAAGGAGTTCA
ACCTCTCACTGGCCGTGAAGCACAATACCATCACGAACGGTCTGAAGTACTCGCTCGCCACAGGAAACTGGGGTGACCAGAAGAAGATGATGTCCGCGAAGGCCGGTGTCTC
TCAAGTGCTGAACAGGTACACATACGCTTCTACCCTGTCGCATCTGCGTCGGTGTAATACACCGTTGGGTCGCGAGGGCAAGATCGCGAAGCCCCGTCAATTGCACAACACGC
ATTGGGGGATGGTGTGCCCCGCCGAGACGCCAGAAGGACAGGCTTGTGGTCTCGTCAAGAACTTGTCGCTCATGTCGTGTATCTCCGTCGGTTCGCTCTCTGCGCCCGTCATT
GAGTTCCTGGAGGAGTGGGGGCTGGAGTCGCTCGAGGAGAACGCACACTCGTCGACACCATGCACAAAGGTGTTCGTCAACGGCGTGTGGATGGGCGTGCACCGCGACCCC
GCGAACTTGGTCAAGACGATCAAGAAGCTGCGGCGCAAGGACGACATCTCGCCCGAGGTGTCCGTCGTGCGCGACATCCGCGAGCGCGAACTGCGTCTGTACACGGACGCC
GGGCGCGTATGCCGGCCGCTCTTCATCGTCGAGAACCAGCAGCTC

> *T. andreanae*-tef1

TCATCGCCGCCGGCACTGGTGAGTTCGAGGCCGGTATCTCCAAGGACGGTCAGACTCGCGAGCACGCTCTGCTTGCCTTCACCCTCGGTGTCAGGCAGCTCATCGTCGCCATC
AACAAGATGGACACCACCAAGGTAAGGGCCTCTTTGTCGTGTTCCGTCGATCAGTCGCTGACGATCTCTGCAGTGGAGCGAGGACCGCTTCAACGAAATCGTTAAGGAGACG
TCCACCTTCATCAAGAAGGTCGGTTACAACCCCAAGTCCGTCGCGTTCGTGCCCATCTCGGGCTGGCACGGCGACAACATGTTGGAGGAGTCTGCCAAGTGAGTATATGCGCT
TTATTCCGCGATTTTCTCGTCCTGATCCTCCATTCTTAGCATGACCTGGTACAAGGGTTGGACCAGGGAGACCAAGGCGGGTGTTGTTAAGGGCAAGACGCTGCTCGATGCCA
TCGATGCCATCGAGCCGCCAGCGCGTCCCTCCGACAAGCCCCTCCGTCTCCCCCTCCAGGATGTCTACAAGATTGGCGGTATCGGCACGGTGCCCGTCGGTCGTGTTGAGACT
GGTATCATCAAGGCCGGCATGGTCGTCACCTTCGCCCCCGTCGGTGTGACCACTGAAGTCAAGTCCGTCGAGATGCATCACGAGCAGCTCGAGCAGGGTCTCCCCGGTGACA
ACGTCGGCTTCAACGTCAAGTGAGTGCCTCTTCGTCTTTCGCTGCGTCGTATACCAATCGTATTTTCAGGAACGTGTCCGTTAAGGATATCCGCCGTGGCAACGTCGCTTCCGA
CTCGAAGAACGACCCCGCCAAGGAGGCCGCGTCCTTCAACGCTCAGGTCATTATCCTGAACCACCCTGGTCAGATCGGTGCCGGCTACGCCCCCGTCCTCGATTGCCACACTG

CCCACATTGCCTGCAAGTTCGCCGAGCTCATCGAGAAGATCGATCGTCGTACCGGCAAGTCGATCGAGGCGGCGCCCAAGTTCGTCAAGTCTGGTGATGCATGCATTGCCAA
GCTCGTCCCCAGCAAGCCCATGTGTGTCGAGGAGT

Table S1. Sequences used in the multilocus phylogenetic tree

Fungal species	Sample no.	Accession no.					Origin	References
		ITS	28S	rpb1	rpb2	tef1		
Ceriporiopsis gilvescens	Chen 3340	MZ636936	MZ637099	MZ748446	OK136039	MZ913651	Taiwan	Chen et al. (2021)
Ceriporiopsis kunmingensis	CLZhao 152	KX081072	KX081074	–	–	–	China: Yunnan	Zhao and Wu (2017)
Ceriporiopsis kunmingensis	CLZhao 153 (holotype)	KX081073	KX081075	–	–	–	China: Yunnan	Zhao and Wu (2017)
Mycoacia aurea	RLG-5075-Sp	KY948759	MZ637161	KY948918	–	MZ913720	USA: New York	Justo et al. (2017); Chen et al. (2021)
Stereophlebia tuberculata	Chen 3242	MZ637088	MZ637285	–	–	–	Taiwan	Chen et al. (2021)
Stereophlebia tuberculata	Wu 1708-107	MZ637089	MZ637286	MZ748450	OK136042	MZ913660	China: Liaoning	Chen et al. (2021)
Phlebia lividina	HHB-4160-Sp	KY948755	KY948849	KY948916	OK136041	MZ913659	USA: North Carolina	Justo et al. (2017); Chen et al. (2021)
Lilaceophlebia livida	FBCC937	LN611122	LN611122	–	–	–	Finland	Kuuskeri et al. (2015)
Lilaceophlebia sp.	FP-135046-Sp	KY948758	KY948850	KY948917	OK136040	MZ913658	USA: Montana	Justo et al. (2017); Chen et al. (2021)
Mycoacia subfascicularis	Wu 1004-11	MZ637008	–	MZ748448	OK136044	MZ913653	Taiwan	Chen et al. (2021)
Mycoacia fuscoatra	GC 1705-1	MZ637004	MZ637165	MZ748447	OK136043	MZ913652	Taiwan	Chen et al. (2021)
Mycoacia nothofagi	GC 1805-5	MZ637005	MZ637166	MZ748449	–	–	Taiwan	Chen et al. (2021)
Fomitopsis pinicola	AFTOL-770	AY854083	AY684164	AY864874	AY786056	AY885152	Unknown	Lutzoni et al. (2004)
Daedalea quercina	FP-56429	KY948809	KY948883	KY948989	–	–	USA: Pennsylvania	Justo et al. (2017)

Sources:

Chen, C. C., Chen, C. Y., and Wu, S. H. (2021). Species diversity, taxonomy and multi-gene phylogeny of phlebioid clade (*Phanerochaetaceae*, *Irpicaceae*, *Meruliaceae*) of *Polyporales*. Fungal Divers. 111, 337–442. doi:10.1007/s13225-021-00490-w.

Justo, A., Miettinen, O., Floudas, D., Ortiz-Santana, B., Sjökvist, E., Lindner, D., et al. (2017). A revised family-level classification of the *Polyporales* (*Basidiomycota*). Fungal Biol. 121, 798–824. doi:10.1016/j.funbio.2017.05.010.

Kuuskeri, J., Mäkelä, M. R., Isotalo, J., Oksanen, I., and Lundell, T. (2015). Lignocellulose-converting enzyme activity profiles correlate with molecular

systematics and phylogeny grouping in the incoherent genus *Phlebia* (*Polyporales*, *Basidiomycota*) Ecological and Evolutionary Microbiology. BMC Microbiol. 15. doi:10.1186/s12866-015-0538-x.

Lutzoni, F., Kauff, F., Cox, C. J., McLaughlin, D., Celio, G., Dentinger, C., et al. (2004). Assembling the fungal tree of life: Progress, classification, and evolution of subcellular traits. *Am. J. Bot.* 91, 1446–1480. doi:10.3732/ajb.91.10.1446.

Zhao, C. L., and Wu, Z. Q. (2017). *Ceriporiopsis kunmingensis* sp. nov. (Polyporales, Basidiomycota) evidenced by morphological characters and phylogenetic analysis. *Mycol. Prog.* 16, 93–100. doi:10.1007/S11557-016-1259-8.
